# Role of PNPLA3 in Hepatic Stellate Cells and Hepatic Cellular Crosstalk

**DOI:** 10.1111/liv.16117

**Published:** 2024-10-12

**Authors:** Maria Castanho Martins, Emmanuel Dauda Dixon, Giulia Lupo, Thierry Claudel, Michael Trauner, Krista Rombouts

**Affiliations:** ^1^ Regenerative Medicine and Fibrosis Group, Institute for Liver and Digestive Health University College London, Royal Free Campus London UK; ^2^ Hans Popper Laboratory of Molecular Hepatology, Department of Internal Medicine III Medical University of Vienna Vienna Austria

**Keywords:** hepatic stellate cells (HSCs): patatin‐like phospholipase domain containing 3 (PNPLA3), liver cirrhosis, liver fibrosis, liver inflammation, metabolic dysfunction‐associated steatohepatitis (MASH), metabolic dysfunction‐associated steatotic liver disease (MASLD), nuclear receptors (NR)

## Abstract

**Aims:**

Since its discovery, the patatin‐like phospholipase domain containing 3 (PNPLA3) (rs738409 C>G p.I148M) variant has been studied extensively to unravel its molecular function. Although several studies proved a causal relationship between the PNPLA3 I148M variant and MASLD development and particularly fibrosis, the pathological mechanisms promoting this phenotype have not yet been fully clarified.

**Methods:**

We summarise the latest data regarding the PNPLA3 I148M variant in hepatic stellate cells (HSCs) activation and macrophage biology or the path to inflammation‐induced fibrosis.

**Results:**

Elegant but contradictory studies have ascribed PNPLA3 a hydrolase or an acyltransferase function. The PNPLA3 I148M results in hepatic lipid accumulation, which predisposes the hepatocyte to lipotoxicity and lipo‐apoptosis, producing DAMPs, cytokines and chemokines leading to recruitment and activation of macrophages and HSCs, propagating fibrosis. Recent studies showed that the PNPLA3 I148M variant alters HSCs biology via attenuation of PPARγ, AP‐1, LXRα and TGFβ activity and signalling.

**Conclusions:**

The advent of refined techniques in isolating HSCs has made PNPLA3's direct role in HSCs for liver fibrosis development more apparent. However, many other mechanisms still need detailed investigations.

**Abbreviations**
8‐iso‐PGF2α: 8‐isoprostaglandin F2α
αSMA: alpha smooth muscle actin
AP1: activator protein 1
ARG1: arginase 1
ASO: antisense oligonucleotide
CCL5: chemokine (C‐C motif) ligand 5
ChREBP: carbohydrate response element binding protein
COX‐2: cyclooxygenase 2
CRBP‐I: cellular retinol‐binding protein type I
CXCL8: chemokine (C‐X‐C motif) ligand 8
CYGB: cytoglobin
DAMPs: danger‐associated‐molecular‐pattern molecules
DGAT1: diacylglycerol O‐acyltransferase 1
DR5: death receptor 5
ECM: extracellular matrix
EGFR: epidermal growth factor receptor
ERα/NR3A1: oestrogen receptor‐α
FFA: free fatty acids
GalNac: N‐acetylgalactosamine
GM‐CSF: granulocyte‐macrophage colony‐stimulating factor
HDL: high density lipoproteins
HIF1α: hypoxia‐inducible factor 1 alpha
hPSC: human pluripotent stem cell
HSCs: hepatic stellate cells
IL6: interleukin 6
IRF4: IFNα regulatory factor
ISR: integrated stress response
LPS: lipopolysaccharide
LRAT: lecithin retinol acyltransferase
LXRα: liver X receptor alpha/NR1H3
MASH: metabolic dysfunction‐associated steatohepatitis
MASLD: metabolic dysfunction‐associated steatotic liver disease
MHC‐II: major histocompatibility complex class II receptor
MMPs: matrix metalloproteases
MTCO1/MTCO2: mitochondrially encoded cytochrome C oxidase I and II
NF‐Kb: nuclear factor kappa‐light‐chain enhancer of B‐cell
NGS: next generation sequencing
NR4A1/Nurr77: nuclear receptor subfamily 4 group A member 1
NRF2: nuclear factor erythroid 2‐related factor
OCR: oxygen consumption rate
PNPLA3: patatin‐like phospholipase domain containing 3
PPARγ: peroxisome proliferator‐activated receptor gamma/NR1C3
PPARδ/NR1C2: peroxisome proliferator activated receptor delta
PUFA: poly‐unsaturated fatty acids
RARα/NR1B1: retinoic acid receptor alpha
ROS: reactive oxygen species
RXRα/NR2B1: retinoid X receptor alpha
siRNA: small‐interfering RNA
sNOX2‐dp: soluble NOX2‐derived peptide
SOD2: superoxide dismutase 2
SREBP‐1: sterol regulatory element binding transcription factor 1
SREBP‐1c: sterol regulatory element binding protein‐1c
STAT3: signal transducer and activator of transcription 3
TGF‐β1: transforming growth factor beta 1
TRAIL: tumour necrosis factor‐related apoptosis‐inducing ligand
VARS2: the mitochondrial valyl tRNA synthetase
VDR/NR1I1: vitamin D receptor
VLDL: very low density lipoproteins
Yap: Yes1 associated transcriptional regulator

**Summary**

- This review first describes the hepatic stellate cells (HSCs) with their lipids and vitamin A metabolism and how the vitamin A homeostasis and its retinol esterification is regulated by the patatin‐like phospholipase domain containing 3—PNPLA3 gene, an important enzyme that has been identified to hydrolyse retinyl esters in HSCs.
- It then further describes the impact of the PNPLA3 I148M single nucleotide polymorphism on intercellular crosstalk and dysregulated mechanisms between HSCs, hepatocytes and macrophages promoting liver fibrosis and liver inflammation during metabolic dysfunction‐associated steatotic liver disease (MASLD).

## 1 Introduction

The liver is a key organ involved in homeostatic processes ranging from lipid and glucose metabolism [1] to inflammation [2] and detoxification [3]. As such, the liver has the unique capacity to heal and regrow after injuries as extreme as hepatectomy [4]. The hepatic wound healing response is driven by inflammation and extracellular matrix (ECM) deposition including collagen [5]. To this aim, a network of cooperating cells is required. In the liver, damaged cells, mostly hepatocytes and cholangiocytes, recruit and activate Kupffer cells, monocyte‐derived macrophages, T cells, endothelial cells and hepatic stellate cells (HSCs) [6]. However, even a robust response can become maladaptive, especially during chronic liver injury as seen with metabolic dysfunction‐associated steatotic liver disease (MASLD) resulting in fibrosis as key step on the road to advanced chronic liver disease and cancer [7].

Liver fibrosis is characterised by an excessive accumulation of interstitial and fibrillar ECM including types I, III and IV collagens, fibronectin, laminin and proteoglycans after persistent inflammatory assault in the liver [8, 9]. Fibrosis disrupts the normal liver architecture and eventually evolves into cirrhosis, characterised by fibrotic bands, a ring of scar around parenchymal nodules and vascular distortion, leading to liver cell dysfunction, portal hypertension and hepatocellular carcinoma [10, 11]. Although early fibrosis can have reversible components [12, 13], later stages with more profound architectural changes become an irreversible condition [14]. Danger‐associated‐molecular‐pattern molecules (DAMPs) released from damaged hepatocytes or cholangiocytes and proinflammatory cytokines and chemokines released by HSCs promote trans‐differentiation and/or myofibroblast activation [15], in addition to genetic factors [16]. The fate of the fibrotic liver to either morph into an anti‐fibrotic scar‐dissolving stage or proceed to a fibrosis‐promoting stage is mainly regulated by resident and infiltrating immune cells, hepatocytes and HSCs. The apoptotic hepatocyte‐released DAMPs and the proinflammatory cytokines and chemokines secreted by HSCs elicit the recruitment and activation of immune cells to activate HSCs and promote trans‐differentiation and myofibroblast activation [8, 15, 17]. Therefore, the intercellular cross‐communication between parenchyma and non‐parenchyma cells is crucial to understand the pathogenesis of liver fibrosis.

## 2 Hepatic Stellate Cells—Historical Perspective, Lipid and Vitamin A Metabolism and PNPLA3

Hepatic stellate cells, first described by Carl von Kupffer in 1876 as ‘Sternzellen’ was based on gold chloride staining of vitamin A‐containing droplets. The correlation between vitamin A level in HSCs and liver fibrosis was first demonstrated by Hans Popper [18], considered by many as the founding father of hepatology. Various staining techniques, like the Golgi silver method, the fat‐staining method used by Ito and Nemoto [19], the vitamin A autofluorescence and electron microscopy by Wake [20], Geerts [21] and the silver impregnation technique, were used to characterise HSCs. By the mid‐20th century, its role in liver injury and fibrosis became more apparent after the refinement of methods for HSCs isolation and characterisation. Scott Friedman discovered HSCs as the source of fibrosis, and described the first HSCs isolation technique, based on an in situ digestion followed by density gradient centrifugation based on the presence of intracellular vitamin A lipid droplets [22, 23]. Other approaches include fluorescent cell sorting based on endogenous vitamin A fluorescence and specific markers [24, 25] and explant culture [26]. In 1971, thanks to the combined work of multiple investigators, HSCs were established as liver‐specific pericytes of non‐parenchymal type and thus clearly distinguished from other non‐parenchymal cells such as Kupffer cells as the resident macrophages, Pit cells or natural killer (NK) cells and endothelial cells. In the human liver, the ratio of HSCs to hepatocytes is approximately 1:10. HSCs are localised in the space of Disse between the basolateral surface of the hepatocytes and the anti‐luminal side of sinusoidal endothelial cells. HSCs are smaller in size compared to hepatocytes and have an average nucleus‐to‐nucleus distance of 40 μm with the presence of long processes, which can be up to 140 μm long and run in parallel to the sinusoidal endothelial wall, thus making contact with endothelial cells, hepatocytes, Kupffer cells, neighbouring HSCs and with nerve endings [21].

In their quiescent form in healthy liver, HSCs store vitamin A in lipid droplets. HSCs in normal healthy liver contain up to 70%–95% of all retinoid storage of the body, derived from the dietary intake released into the bloodstream as retinyl‐esters containing chylomicron remnants taken up by the hepatocytes [27]. In diseased liver, activated HSCs loose these retinyl ester stores, ultimately leading to vitamin A deficiency [28]. Therefore, understanding the mechanisms behind the retinol transfer between hepatocytes and HSC and retinol loss in activated HSCs is physiologically vital. Vitamin A storage and homeostasis is a complex process that orchestrates a fine balance between enzymes, such as the acetyltransferases and the hydrolases, to maintain the HSCs in a quiescent state in a healthy liver. Investigators have demonstrated that retinoic acid (RA) is released from retinyl ester stored in HSCs during the initial phase of HSCs activation through RXR‐JNK‐AP‐1‐mediated pathways [29, 30]. Nevertheless, under normal healthy conditions, over 95% of the retinoids are stored as retinyl esters in cytoplasmic perinuclear lipid droplets [31]. The droplets are electron‐dense, with‐ or without a membrane unit (i.e., type I and type II lipid droplets, respectively), and have variable size and content depending on HSCs subpopulations and activation. Whether these different types of lipid droplets are related to the heterogeneous HSCs population has not been investigated yet. Nevertheless, when performing single‐cell RNA sequencing on primary human HSCs, Payen et al. have demonstrated that HSCs in the human liver are heterogeneous, spatially zoned and characterised by unique gene expression signatures suggestive of crucial functional differences [32]. Thus, until today, our knowledge concerning the vitamin A uptake, origin and relationship between the two types of lipid droplets remains limited [33, 34, 35]. Upon isolation and purification of primary HSCs, using the autofluorescence of vitamin A‐containing lipid droplets, HSCs cultured on plastic spontaneously become activated into myofibroblast‐like cells, a process marked by loss of lipid droplets, retinyl esters and the enzyme lecithin retinol acyltransferase (LRAT) expression [36]. In contrast to these in vitro observations, in vivo‐activated HSCs do not lose their vitamin A droplets completely [37]. The vitamin A homeostasis and its retinol esterification were thought to be mainly regulated by the enzymatic activity of LRAT. Thus, investigators performed in vivo experiments using LRAT‐deficient mice and surprisingly demonstrated no increase in liver fibrosis but less tumour load in LRAT‐deficient mice. These results indicated that the absence of retinoid‐containing lipid droplets in HSCs does not promote HSCs activation but reduces cancer development [34, 38]. Moreover, LRAT‐deficient mice, lacking the retinyl ester‐containing lipid droplets, demonstrated a delay in normal liver regeneration after hepatectomy [39]. As LRAT is not present in HSCs from LRAT knockout mice, the retinyl esters are synthesised by an alternative pathway, which involves diacylglycerol O‐acyltransferase 1 (DGAT1) enzyme activity, thus allowing HSCs to retain the capacity to synthesise retinyl esters stored in lipid droplet containing different retinyl ester species [40].

Conversely, three enzymes have been identified to hydrolyse retinyl esters in HSCs: adipose triglyceride lipase/patatin‐like phospholipase domain containing 2 (ATGL/PNPLA2), adiponutrin (ADPN/PNPLA3) and hormone‐sensitive lipase (HSL), which are upregulated upon activation in cultured rat HSCs [41]. Interestingly, the PNPLA3 protein has lipase activity towards triglycerides in hepatocytes and retinyl esters in HSCs. However, their lipase activity is lower compared to its homology, PNPLA2 and the hydrolase activity is lost in the I148M variant version of the PNPLA3 through sequestration of CG1‐58 from PNPLA2 [42].

## 3 PNPLA3 and Profibrogenic and Pro‐Inflammatory Action

**FIGURE 1:**
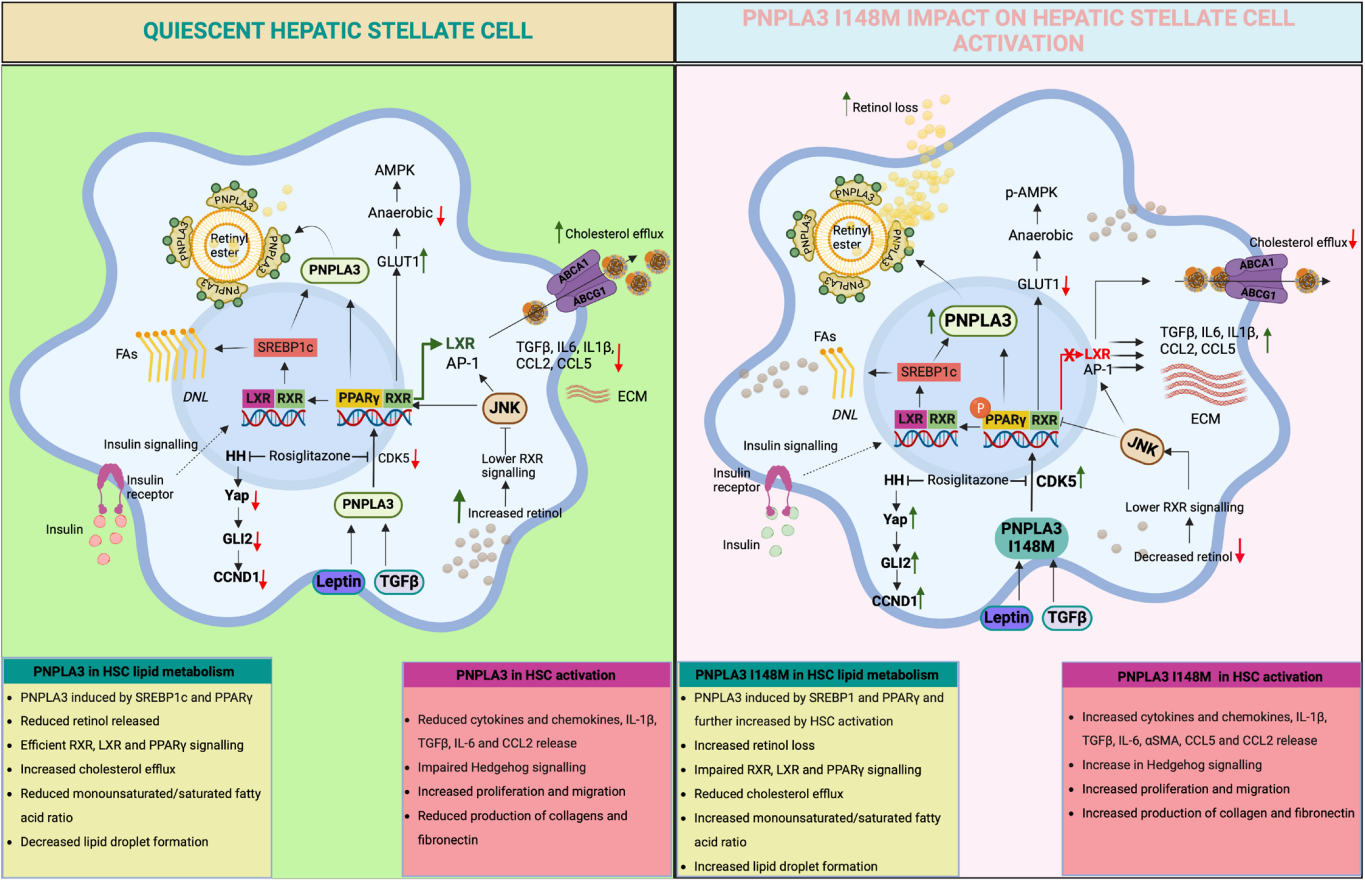
Mechanism of PNPLA3 I148M action in HSC activation. In addition to insulin and SREBP1c, PPARγ transcriptionally regulates the *PNPLA3* gene. Moreover, leptin, TGFβ and HSC activation also stimulate the expression of PNPLA3. In HSCs carrying the *PNPLA3* I148M variant, JNK and AP‐1 activation impairs PPARγ and LXR signalling, initiating the expression of genes involved in inflammation, proliferation, migration and production of ECM. Further, PPARγ phosphorylation via CDK5 (unpublished data), leads to the repression of ABCA1 and ABCG1 and accumulation of cholesterol further driving inflammation and HSC activation. Leptin and TGFβ increase the *PNPLA3* I148M variant and activate the Hedgehog and Yap signalling pathways. Figure was created with BioRender. ABCA1/G1, ATP binding cassette subfamily A member 1/G member 1; AP‐1, activator protein 1; CDK5, cyclin‐dependent kinase 5, ECM, extracellular matrix; HSC, hepatic stellate cell; JNK, c‐Jun‐N‐terminal kinase; LXR, liver X receptor; PNPLA3, patatin‐like phospholipase domain‐containing protein 3; PPARγ, peroxisome proliferator‐activated receptor gamma; SREBP1c, sterol regulatory element‐binding protein 1c; TGFβ, tumour growth factor beta.

HSCs are activated by the release of cytokines/chemokines by platelets and inflammatory cells, by damage‐associated reactive oxygen species (ROS) and the generation of lipid peroxides and apoptotic bodies by damaged hepatocytes [43]. Thus, once activated, HSCs are marked by an increased secretion of factors, in an autocrine manner and in response to the various microcellular—environmental changes [44]. This transition into an activated HSCs is marked by the initiation of changes in gene expression of multiple signalling molecules and pathways [45], with the genetic *PNPLA3* polymorphism I148M exacerbating the development and progression of MASLD towards MASH. PNPLA3 is highly expressed in human HSCs compared to hepatocytes (higher in primary HSCs compared to hepatocytes) [29, 30] and is influenced by nutritional status [46, 47]. *PNPLA3* gene and protein expression significantly increases during the early phases of HSCs activation and remains elevated in fully activated HSCs in vitro, indicating that PNPLA3 is required for HSCs activation [30]. A single nucleotide polymorphism (rs738409; C>G) in the *PNPLA3* gene encoding the I148M variant heightens the risk of fibrosis [16]. In liver biopsies of MASH patients, PNPLA3 directly correlated with fibrosis stage and relative quantification of smooth muscle actin (αSMA), independent of the genotype [47]. PNPLA3 is regulated transcriptionally by insulin through the induction of Sterol Regulatory Element Binding Protein‐1c (SREBP‐1c) and carbohydrate response element binding protein (ChREBP) [48]. As mentioned before, in HSCs, PNPLA3 catalyses the hydrolysis of retinyl esters. In vitro and *ex vitro* studies have shown that HSCs carrying the *PNPLA3* I148M variant retain retinol due to lack of hydrolase activity. In line, human liver tissue from *PNPLA3* I148M homozygous variants demonstrated higher retinol concentrations. Furthermore, the effect of PNPLA3 on fibrosis severity among patients with different liver diseases has been established, with the presence of the SNP leading to the activation of HSCs [49, 50]. However, until recently, the mechanistic relations between PNPLA3, inflammation and fibrosis were unclear. Among the three PPAR isotypes, PPARγ (Peroxisome Proliferator‐Activated Receptor gamma/NR1C3) was identified as a key nuclear receptor expressed in quiescent HSCs. Its expression was diminished upon activation when concomitantly AP‐1 and NF‐KB activities increased [51]. Recent advances established that HSCs carrying *PNPLA3* I148M had reduced expression of PPARγ due to increased JNK activity which phosphorylates PPARγ making it less active together with AP‐1 induction, resulting in cytokine secretion, cell migration and proliferation [30]. In line, the activity of the nuclear receptor LXRα (liver X receptor alpha/NR1H3), a downstream target of PPARγ [52, 53], was also reduced in *PNPLA3* I148M expressing HSCs and in a stable over‐expressing cell line [54]. Impaired LXRα signalling resulted in cholesterol accumulation, limited de novo lipogenesis via SREBP1c downregulation, and exacerbated collagen and α‐SMA production, therefore increasing fibrosis (Figure 1) [54]. Furthermore, transforming growth factor (TGF‐β1) stimulation of HSCs carrying *PNPLA3*‐I148M increased *PNPLA3* expression and this coincided with a reduction in lipid droplets in primary human HSCs [55]. Both the Yes1 Associated Transcriptional Regulator (Yap)—Hippo pathway and the Hedgehog pathway (HH) have been implicated in the HSCs activation process driven by TGF‐β1. Activated HSCs carrying *PNPLA3* I148M showed increased activation of the HH pathway and its downstream effector, Yap, in contrast to wild type PNPLA3 HSCs. Further, when exposed to TGF‐β1 and leptin, total Yap increased rapidly which could be inhibited by the Yap‐specific inhibitor Verteporfin in combination with Rosiglitazone, a PPARγ synthetic agonist (Figure 1) [56]. Indeed, performing next generation sequencing (NGS) and Nanostring Technologies nCounter Human Fibrosis 700 genes panel highlighted pathways of differential expression between *PNPLA3* WT and *PNPLA3* I148M HSCs such as ‘Collagen or ECM Biosynthesis, and modification’ and ‘Hippo Pathway’, ‘TGFB1 signalling’, lipid metabolism (‘PPAR Signalling’, ‘De novo lipogenesis’ and ‘Fatty Acid metabolism’) as well as ‘Oxidative stress’ [57].

Vitamin A is a precursor of retinoids and it is known that retinoids can activate nuclear receptors such as the Retinoic Acid Receptor alpha (RARα/NR1B1) and the Retinoid X Receptor alpha (RXRα/NR2B1) [58]. RXRα can form heterodimers with class II nuclear receptors such as LXRα, PPARγ, RARα [59] or the Vitamin D Receptor (VDR/NR1I1) [60] all expressed in HSCs. However, heterodimers have different activities depending on the ligands of the respective partners. VDR is non‐permissive and as such RXR activation by retinoids impairs the VDR response [61] when PPARs or LXRs heterodimers are activated by retinoids [62]. Important, vitamin A levels in HSCs are controlled by PPARδ (Peroxisome proliferator activated receptor delta/NR1C2). HSCs activation results in increased protein expression of LRAT and CRBP‐I (cellular retinol‐binding protein type I), which expression is further enhanced by PPARδ agonists and inhibited by antisense against PPARδ [63]. Therefore, PPARδ helps to regulate vitamin A levels in HSCs and as such controls RXR activity, its heterodimer partner, as well as the activities of LXR and PPARγ. Interestingly, this control loop impairs VDR activity, which represses cyclin D1, TIMP‐1 and Collagen 1a1, but increases MMP9 in HSCs and thus represses HSCs activation and fibrogenesis [60]. Further, dietary vitamins A and D3 are carried by chylomicrons and their remnants are partitioned into HSCs and adipocytes, respectively. Therefore, PPARδ acts as a crossroad controller to coordinate multiple nuclear receptor pathways via their ligands to fine‐tune HSCs activation. Hence, such a complex system shows how tightly HSC function must be maintained by nuclear receptors in healthy liver. This opens important novel therapeutic opportunities with the nuclear receptor ligands which are now available for the treatment of MASLD [64]. A notable example is the recently approved Resmetirom, a thyroid hormone receptor beta, THRβ, shown to improve moderate to advanced hepatic fibrosis [65].

## 4 PNPLA3 and Mitochondrial Dysfunction—Oxidative Stress and Anti‐Oxidative Stress Response

Liver fibrosis is marked by increased oxidative stress [43] and patients carrying the *PNPLA3* I148M variant demonstrated an increase in systemic oxidative stress as quantified in serum levels of soluble NOX2‐derived peptide (sNOX2‐dp) and 8‐isoprostaglandin F2α (8‐iso‐PGF2α) [66]. At the cellular level, investigators have measured the mitochondrial respiratory activity, by quantifying the Oxygen Consumption Rate (OCR), and found a change in ATP production and mitochondrial membrane potential. This resulted in elevated ROS levels which caused mitochondrial structural damage, an alteration in the oxygen consumption rate (OCR), and a decreased expression of mitochondrial function‐related proteins both in LX2 carrying *PNPLA3* I148M [56, 67] and primary human HSCs [57]. Indeed, VARS2 protein, the mitochondrial valyl tRNA synthetase, a key enzyme in the synthesis of the mitochondrial DNA‐encoded subunits of the respiratory chain enzyme complexes such as complex IV, Mitochondrially Encoded Cytochrome C Oxidase I (MTCO1) and MTCO2, are significantly downregulated in HSCs carrying the *PNPLA3* I148M variant with a significant reduced Complex IV enzymatic activity in *PNPLA3* I148M cells. Therefore, even when the basal respiration was similar between wild type or *PNPLA3* I148M carrying HSCs, the *PNPLA3* I148M‐driven mitochondrial dysfunction in HSCs is not caused due to a lower mitochondrial number but because of a deficiency in Complex IV expression and activity [57]. However, whether these observed effects on mitochondrial dysfunction and VARS2 depletion are linked to activation of the Integrated Stress Response (ISR) in activated *PNPLA3* I148M carrying HSCs needs further research.

Thus, activated HSCs, carrying *PNPLA3* I148M, have impaired mitochondrial function which further leads to diminished oxidative capacity and coincides with a significant reduced antioxidant defence by proteins such as Cytoglobin (CYGB), an oxygen transporter, and nuclear factor erythroid 2‐related factor (NRF2), a key antioxidant enzyme, resulting in enhanced generation of ROS. Furthermore, Superoxide Dismutase 2 (SOD2), the mitochondrial‐specific superoxide dismutase, but not SOD1, was significantly decreased in *PNPLA3* I148M HSCs and a variety of mitochondrial proteins [57, 67]. As a result of the mitochondrial dysfunction and downregulation of important antioxidant enzymes, HSCs carrying *PNPLA3* I148M also showed a significant increase in the formation of ROS species released, such as lipid peroxidation products 4‐Hydroxynonenal (4‐HNE) [57].

## 5 Impact of PNPLA3 on the Crosstalk Between Hepatocyte and HSCs in Fibrogenesis

Hepatocytes are the predominant liver cell mass (about 80%) and perform most liver‐associated functions. As the principal constituent of the liver cell mass, hepatocytes receive initial lipid insults from the peripheral tissues, like the adipose tissue and sense fatty acids (FA) after being stored as triglycerides in lipid droplets [68] via PPARα (peroxisome proliferator activated receptor alpha/ NR1C1) or as free fatty acids (FFA) via PPARδ [69]. FFA in excess can induce endoplasmic reticulum (ER) stress and mitochondria dysfunction, after TNFα release [70, 71] and tumour necrosis factor‐related apoptosis‐inducing ligand (TRAIL) [72] and its cognate death receptor 5 (DR5) induction [73]. Additionally, FFAs have been shown to trigger the intrinsic apoptosis pathway via JNK, orchestrated by intracellular Bim levels and Bax activation, leading to mitochondrial permeabilisation, cytochrome c release and caspase activation [74]. As key genetic factors contribute to hepatotoxicity, the PNPLA3 genetic variant is linked not only to higher risk for benign steatosis, but also MASH, fibrosis, cirrhosis and end‐stage HCC [16]. Furthermore, human hepatocytes carrying the *PNPLA3* I148M variant have reduced very low density lipoproteins (VLDL) secretion, suggesting that the genetic variant promotes retention of fat instead of apo B lipidation and thus points towards a loss of function [75]. However, these findings were challenged recently: Despite a threefold higher presence in the liver of patients carrying the *PNPLA3* I148M variant, there was no detectable change in VLDL1 nor any other lipid fraction in a population of Finnish men [76]. Since VLDL1 synthesis and secretion by the liver increases directly with liver fat content in obese patients [77] this result was unexpected. Other investigations revealed that I148M *PNPLA3* carriers had increased retention of poly‐unsaturated fatty acids (PUFA) in the liver [78]. Interestingly, PUFA‐phosphatidylcholine (PUFA‐PC) plays a key role during VLDL assembly by promoting a monolayer formation around the neutral core which is then incorporated into nascent VLDL particles and lack of PUFA‐PC leads to the degradation of the VLDL [79]. Taken together, these results could suggest that the increase of VLDL synthesis due to hepatic fat accumulation in *PNPLA3* I148M might be counteracted by the PUFA‐PC deficiency leading to no net difference in VLDL secretion in I148M fatty liver patients. These results were confirmed and PNPLA3 was identified as a lipase hydrolysing PUFA in triglycerides, with the wild type regulating the balance between liver fat storage and secretion, making the *PNPLA3* I148M mutation a loss of function [80]. More interesting results were found in insulin‐resistant *PNPLA3* I148M carriers, who displayed an anti‐atherogenic lipid profile characterised with less and smaller VLDL, less and larger LDL (low density lipoproteins) together with increased high density lipoproteins (HDL) particles [81]. Based on the fact that PNPLA3 increased in the liver of I148M carriers [47, 82] and that antisense oligonucleotides were shown to reduce its expression in transgenic mice [83], it is very likely that such therapies will be tested in humans, as seen with PCSK9 [84] or apo CIII [85]. However, such strategy might have risks since lowering *PNPLA3* I148M in the liver and thus reducing fat depot, might promote a pro‐atherogenic profile translating into exacerbated atherosclerotic risk. Clinical studies will have to adequately address the risk of such dissociation between liver and cardiovascular health.

During fibrosis development in MASLD patients, hepatocytes display a loss of their identity characterised by the appearance of a network of transcription factors activated by fibrotic stimuli such as transcription factor Elf‐3 (ELF3) and zinc finger protein GLIS2 (GLIS2). This in turn reprogrammes hepatocyte's identity and leads to a vicious cycle of cytokines/hepatokines/stellakines modifying cellular activities in the liver, explaining how dysfunctional hepatocytes and HSCs drive fibrosis [86]. In line with these data, it was shown that vitamin A was shifting from HSCs towards hepatocytes during MASLD development in mice and in vitro upon palmitic acid incubation [87], although the direct impact on PNPLA3 was not studied.

When using multilineage 3D spheroids composed by hepatocytes (HepG2) and hepatic stellate cells (LX‐2), both cell types carrying *PNPLA3* I148M, treatment with free fatty acids such as palmitic acid and oleic acid resulted in significant increase in lipid accumulation and collagen 1a1 expression. The impact of free fatty acid exposure was rescued by incubation with drugs such as liraglutide or elafibranor, but not by vitamin E or obeticholic acid [88]. Moreover, exposure of these multilineage 3D spheroids to oestrogen receptor‐α (ERα/NR3A1) agonists showed an induction in *PNPLA3* expression which further demonstrated how the interaction between ERα and *PNPLA3* I148M‐carrier hepatocytes can drive fatty liver disease susceptibility in women. Furthermore, when treating the 3D spheroids with free fatty acids, TGF‐β1 or tamoxifen, the latter showed an increase in collagen‐1a1 synthesis and *PNPLA3* mRNA levels. Thus ER‐α‐induced upregulation of *PNPLA3* triggers lipid accumulation in hepatocytes followed by HSCs activation via an ER‐α‐binding site within a PNPLA3 enhancer. As a result, this induced lipid droplet accumulation and fibrogenesis in three‐dimensional multilineage spheroids with HSCs [89]. Further, when using a heterocellular spheroid system containing primary human hepatocytes co‐cultured with a crude mix of primary human liver non‐parenchymal cells, a more fibrotic phenotype was observed in *PNPLA3* I148M donors. These specific donor co‐cultures showed an increased incorporation of vimentin‐expressing HSCs and a higher baseline of extracellular fibrillary matrix [90]. A human pluripotent stem cell (hPSC)‐derived multicellular liver culture system containing hPSC‐derived hepatocytes, HSCs and macrophages and exposed to a lipotoxic milieu containing glucose, insulin, palmitic acid and oleic acid, that is, mimicking risk factors in NAFLD patients showed an increased expression in HSCs activation markers in those HSCs carrying *PNPLA3* I148M with elevated levels of IL6/STAT3, which coincided with a reduction in retinol content and quiescence marker PPARγ [91], as shown before [30]. The comparison of transcriptomic analysis between liver biopsies of obese individuals and in vitro‐cultured primary human HSCs, both genotyped for the presence of *PNPLA3* SNP, demonstrated shared *PNPLA3* I148M‐driven dysregulated pathways related to ECM remodelling and TGF‐β1 signalling, thus showing a major impact of the *PNPLA3* I148M variant on the fibrogenic phenotype of HSCs. Moreover, TGF‐β1 secreted by activated HSCs and known to activate quiescent HSCs, was identified by the transcriptomic data as a key activated upstream regulator—showing increased signalling in *PNPLA3* I148M HSCs versus *PNPLA3* WT HSCs when both genotyped cells were repopulated in bioengineered 3D human‐derived cirrhotic liver ECM scaffolds compared to healthy scaffolds. Furthermore, the nuclear receptor NR4A1 (Nurr77) was highlighted by NGS as differentially modulated in the *PNPLA3* I148M variant and its reduced expression could be counteracted by treatment with cytosporone B, thus increasing Nur77's endogenous anti‐fibrotic modulatory effect on TGFβ1 [57].

## 6 Impact of PNPLA3 on the Crosstalk Between Macrophages and HSCs in Fibrogenesis

In addition to DAMPs and ROS, hepatocytes also secrete chemokines such as chemokine (C‐C) motif ligand 2 (CCL2) [92], which together promote the recruitment of monocytes into the liver, where they develop into macrophages. The recruited macrophages from bone marrow and the self‐renewing liver resident macrophages, termed Kupffer cells, make macrophages the largest non‐parenchyma cell population and the most heterogeneous group of liver cells.

Hepatic macrophages possess a remarkable plasticity resulting in different phenotypes depending on their microenvironment. Based on the types of surface protein markers and cytokines gene expressions, macrophages are classified into M1, or classically activated macrophages, and M2, or alternatively activated macrophages [93]. Lipopolysaccharides and interferon‐gamma (IFNγ)‐stimulated macrophages or M1 is characterised by the release of TNF‐α, IL1‐β, IL‐6, IL‐12 and IL‐23, promote T_H_‐1 responses and produce high amounts of superoxide anions and oxygen and nitrogen radicals to increase their killing capability [94]. The expression of these proinflammatory cytokines and chemokines is activated by transcription factors such as nuclear factor kappa‐light‐chain enhancer of B‐cell (NF‐Kb), IFNγ regulatory factor (IRF4), hypoxia‐inducible factor 1 alpha (HIF1α) and activator protein 1 (AP1), followed by SREBP‐1, STAT1 and STAT3, triggering the expression of CD80, CD86, CIITA, major histocompatibility complex class II receptor (MHC‐II) and cyclooxygenase 2 (COX‐2) [95]. In contrast to classically activated macrophages, the alternatively activated M2 macrophages (analogous to Th2 T cells) generally produce anti‐inflammatory cytokines like interleukin‐10 (IL‐10) and transforming growth factor‐β (TGFβ) when induced by IL‐4 and IL‐13, both of which inhibit the M1 phenotype to permit resolution of inflammation and tissue repair. However, when injury persists, M2 macrophages assume a pro‐fibrotic role and secrete pro‐fibrotic factors, such as TGF‐β, as often seen in the case of liver fibrosis [96]. They express high levels of mannose receptor (CD206), CD163, PPARγ, STAT6, matrix metalloproteases (MMPs) and arginase 1 (ARG1). The high arginase activity results in the production of polyamines and collagen that favours tissue remodelling and wound healing [97].

**FIGURE 2:**
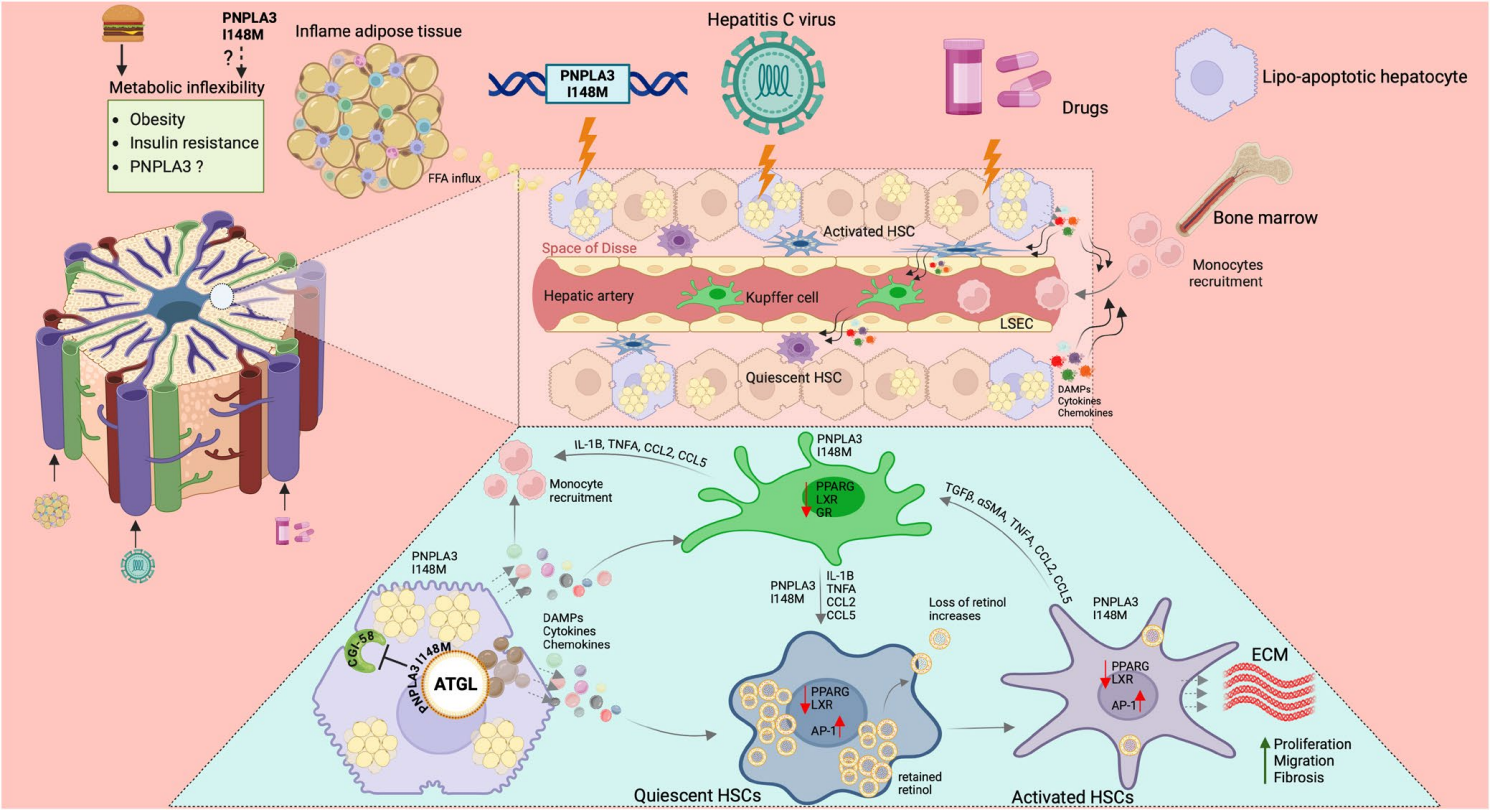
The principal mechanisms of intercellular crosstalk in liver fibrosis. Events that can lead to liver injury and hepatoxicity include systemic dysregulation of energy metabolism characterised by obesity and IR and permit the continuous insults of FFA from WAT to the liver. Viral infection, as in the case of the hepatitis C virus, drugs or the sheer possession of the *PNPLA3* I148M and its ability to sequestrate CGI‐58 access to ATGL can also instigate liver injury hepatotoxicity. The damaged hepatocytes trigger inflammation, which is maladaptive and further instigate tissue repair dominated by the trans‐differentiation of quiescent HSCs into extracellular matrix‐producing myofibroblasts, which drive liver fibrosis and activate the liver resident macrophage Kupffer cells. The activated Kupffer cells and HSCs, in turn, release cytokines and chemokines, which encourage the recruitment of monocytes, further exacerbating the activation of HSCs, characterised by the loss of retinol. Figure was created with BioRender. ATGL; adipose triglyceride lipase, CGI‐58, comparative gene identification‐58; FFA, free fatty acids; HSCs, hepatic stellate cells; WAT, white adipose tissue.

Functionally distinct macrophage subpopulations coexist in the same tissue and play a critical role in the injury and recovery phase of inflammatory scarring. Macrophage depletion in the early phase of liver injury decreases the inflammatory response and reduces scarring and the number of myofibroblasts. In contrast, macrophage depletion during recovery leads to a failure of ECM degradation and a less efficient repair [98]. At the early phase of injury or during the initiation phase, the predominant macrophage populations are the proinflammatory type. The Kupffer cells which become activated or sensitised by the DAMPs such as RNA, DNA or HMBG‐1 and ROS from hepatotoxic and cholangiotoxic cells, rapidly secrete proinflammatory chemokines and cytokines like CCL2, CCL5, IL‐1β and TNFα to activate HSCs and recruit other immune cells including monocyte‐derived macrophages [99, 100]. The recruited immune cells further perpetuate HSCs activation through Toll‐like Receptor 4 (TLR4) activation leading to TGFβ sensitisation and further release of CCL2 [99], thus recruiting more immune cells for the sustained injury leading to advanced fibrosis (Figure 2). Others have shown mutual stimulation and amplification of the fibrogenic response through the epidermal growth factor receptor (EGFR)‐dependent pathway [101] and cadherin‐11 [102] between HSCs and Kupffer cells.

Although hepatic macrophages play a central role in the initiation and progression of various liver diseases including MASLD [103], there have however, to date been no studies on the role of PNPLA3 in macrophages in metabolic liver diseases. Recent data suggest that macrophages carrying the *PNPLA3* I148M variant are proinflammatory further enhancing the inflamed phenotype to dysregulation in lipid metabolism in MASLD (*Dixon et al., in submission*) [104]. More specifically, HSCs carrying *PNPLA3* I148M versus wild type PNPLA3 HSCs demonstrated a significant increase in cytokines such as chemokine (C‐C motif) ligand 5 (CCL5), granulocyte‐macrophage colony‐stimulating factor (GM‐CSF) and chemokine (C‐X‐C motif) ligand 8 (CXCL8). When exposing THP‐1 differentiated macrophages to conditioned media derived from *PNPLA3* I148M or wild type PNPLA3 HSCs, these macrophages demonstrated an enhanced chemotaxis [30]. Indeed, gene expression profiled by next generation sequencing (NGS) and analysed by Ingenuity Pathway Analysis (IPA) highlighted a wide number of significantly deregulated ‘Canonical Pathways’ in primary human HSCs carrying *PNPLA3* I148M ranging from increased fibrogenesis, inflammation, metabolism and proliferation. HSCs carrying *PNPLA3* I148M demonstrated the highest number of divisions when stained by cytopainter and quantified over 7 days of culture [57]. Further, a human in vitro triple cell culture MASH model, with primary human hepatocytes, Kupffer cells and HSCs, was used as microtissues in a perfused three‐dimensional microphysiological system and showed that specific stimuli such as free fatty acids induced a pro‐fibrogenic environment, whereas lipopolysaccharide (LPS) exposure resulted in a proinflammatory milieu. Moreover, HSCs carrying *PNPLA3* I148M caused a proinflammatory milieu with increased expression/secretion of IL‐6 and other proinflammatory cytokines when microtissues were treated with free fatty acids with or without LPS further indicating a strong crosstalk between hepatocytes, Kupffer cells and HSC carrying *PNPLA3* I148M [105].

## 7 Conclusions

The advent of HSCs isolation techniques increased our understanding of HSCs biology, their role in vitamin A storage, ECM remodelling and their importance to liver fibrosis. Since liver fibrosis is the most critical prognostic determinant of survival, it may not be surprising that such an intricate web of cell types, nuclear receptor sensors and lipolytic enzymes control this homeostatic response to maintain metabolic flexibility, which can be compromised by lifestyle and genetic predisposition. The *PNPLA3* I148M variant, independent of its functional annotation with hydrolase activity and acyltransferase, results in hepatic steatosis, which can advance to fibrosis. Importantly, the proinflammatory and profibrogenic role of the *PNPLA3* I148M variant may also be attributed at least in part to its role in non‐parenchyma liver cells such as HSCs and macrophages. With MASLD increasing globally, together with the prevalence of obesity and the interaction between genetic predisposition and other factors partially explaining the large variability observed in MASLD patients' phenotype and natural history, there is a need to further increase our knowledge on the role and mechanisms of PNPLA3 (variants) in MASLD or MASH [106, 107, 108].

Against this backdrop, therapeutic strategies are currently being developed, with silencing of *PNPLA3* using oligonucleotide‐based therapies, namely small‐interfering RNA (siRNA) and antisense oligonucleotide (ASO) in human *PNPLA3* I148M variant knock‐in mouse. The currently used ASOs are N‐acetylgalactosamine (GalNac) conjugated and therefore very specific to the hepatocyte, thus not directly targeting HSCs or other hepatic cell types [109]. Further, it still needs to be shown whether improving hepatocellular lipid metabolism will also translate into a reduction of hepatic fibrosis and that approaches also, if not exclusively, targeting HSCs may still be worthwhile to be considered to treat fibrosis, in non‐MASLD‐related aetiologies and MASLD [83], thus paving the way for precision medicine [110].

## Conflicts of Interest

Michael Trauner received grant support from Albireo, Alnylam, Cymabay, Falk, Genentech, Gilead, Intercept, MSD, Takeda and UltraGenyx, honoraria for consulting from AbbVie, Albireo, Agomab, Boehringer Ingelheim, BiomX, Chemomab, Dexoligo Therapeutics, Falk, Genfit, Gilead, GSK, Hightide, Intercept, Ipsen, Jannsen, MSD, Novartis, Phenex, Pliant, Regulus, Siemens and Shire, speaker fees from Albireo, Boehringer Ingelheim, Bristol‐Myers Squibb, Falk, Gilead, Ipsen, Intercept, MSD and Madrigal, as well as travel support from AbbVie, Falk, Gilead, Jannsen and Intercept. He is also a co‐inventor of patents on the medical use of 24‐norursodeoxycholic acid filed by the Medical University of Graz. Krista Rombouts owns shares or receives stock options in Engitix Therapeutics Ltd. and receives consultancies from Engitix Therapeutics Ltd. All other authors declare no conflicts of interest.
